# The importance of *TP53* status in cancer therapy: The example of chronic lymphocytic leukemia

**DOI:** 10.22099/mbrc.2025.51477.2054

**Published:** 2025

**Authors:** Regina Mirgayazova, Raniya Khadiullina, Elvina Gilyazova, Damir Davletshin, Irina Ganeeva, Ekaterina Zmievskaya, Vitaly Chasov, Aygul Valiullina, Emil Bulatov

**Affiliations:** 1 Institute of Fundamental Medicine and Biology, Kazan Federal University, 420008 Kazan, Russia; 2 Shemyakin-Ovchinnikov Institute of Bioorganic Chemistry, Russian Academy of Sciences, 117997 Moscow, Russia

**Keywords:** Chronic lymphocytic leukemia, TP53 gene, Mutation, CAR-T therapy

## Abstract

The *TP53* gene encodes the tumor suppressor protein p53, which plays a critical role in genomic stability and cell cycle regulation. *TP53* mutations are prevalent in approximately half of all human malignancies and are associated with poor clinical outcomes, including increased genomic instability, chemoresistance, and reduced survival rates. However, the prognostic and predictive value of *TP53* status remains inconsistent across cancer types. Chronic lymphocytic leukemia (CLL) stands out as a disease where *TP53* alterations have a well-established clinical significance, influencing treatment decisions and patient prognosis. In CLL, *TP53* mutations and 17p deletions are strongly correlated with advanced disease stages, resistance to chemo-immunotherapy, and poor overall survival. The European Research Initiative for CLL (ERIC) has recognized *TP53* status as a crucial prognostic biomarker, advocating for its routine assessment in clinical practice. Given the limitations of traditional therapies in *TP53*-mutated CLL, novel targeted therapies, including BCL2 and BTK inhibitors, as well as CAR-T cell therapy, are being explored to improve patient outcomes. This review provides an in-depth analysis of the evolving role of *TP53* status in CLL, with a particular focus on emerging therapeutic strategies, including CAR-T cell therapy, and their potential to overcome *TP53*-driven treatment resistance.

## INTRODUCTION

Analysis of more than 20,000 cancer genomes has identified *TP53* as the most frequently mutated gene in human cancer [1]. The *TP53* gene, located on the long arm of chromosome 17, consists of 11 exons spanning approximately 20,000 base pairs in the genome. The gene encodes a 53 kDa nuclear phosphoprotein of 393 amino acid residues. There are four functional domains that regulate transcription, DNA binding, oligomerisation and autoinhibition [2]. 

*TP53* mutations are frequently found in specific hotspots, particularly within the DNA-binding domain (DBD). However, their prevalence varies significantly across different cancer types. For instance, lung adenocarcinoma and squamous cell carcinoma exhibit distinct mutation frequencies compared to gastrointestinal cancers, largely due to differing mutagenic processes and selective pressures unique to each cancer type. These include exposure to environmental carcinogens such as tobacco smoke and aflatoxins. The functional consequences of *TP53* mutations are diverse and can include loss-of-function (LOF), dominant-negative (DN) effects, or gain-of-function (GOF) activities. Interestingly, no significant gene expression changes have been observed between different mutation types in large tumor cohorts. This may be because the functional impact of these mutations is more critical during the early stages of tumor development and diminishes as tumors progress to clinically detectable stages.

Moreover, *TP53* mutations influence transcriptional regulation in a tissue-specific manner, which may not be fully captured in broad transcriptomic analyses. These mutations also alter the tumor microenvironment (TME), with varying effects across cancer types. For example, reduced CD8+ T cell infiltration has been observed in head and neck squamous cell carcinoma (HNSC) and uterine corpus endometrial carcinoma (UCEC), which may affect the response to immune checkpoint inhibitors. Thus, *TP53* mutations exhibit complex, context-dependent effects, contributing to the variability and inconsistency in their clinical significance across different cancers [3].

Despite the fact that *TP53* is altered in more than 50% of human tumors, its prognostic significance in various cancers remains uncertain, and the results of studies on this topic have occasionally been contradictory [4-6]. Possible factors contributing to these inconsistent findings include the methodology and strategy used to determine *TP53* status, the diversity of tumor types, tumor genetics, and the prevalence of *TP53* mutations.

To mitigate these factors, all *TP53* variants were classified according to the type of mutation (missense or indel), the protein's intracellular location, and the long-term stability of the altered residue. This classification has improved the clinical value of *TP53* status in head and neck cancer [7], breast cancer [8] or diffuse large B-cell lymphoma [9]. However, a clear algorithm and rationale for the definitive assessment of *TP53* alterations is still lacking.

There is no doubt that mutant *TP53* acts as an oncogene and leads to tumor progression. However, there are still many open questions: in which cancer types/subtypes will the determination of *TP53* status be of greatest benefit; what is the contribution of *TP53* variant heterogeneity to tumor phenotype; is the contribution of *TP53* isoforms to tumor phenotype large; which drugs will be most effective in tumors with functional and non-functional *TP53* pathways [10].

Although the prevalence of *TP53* gene alterations in asymptomatic individuals is relatively low, chronic lymphocytic leukemia is currently the best-known example of the clinical significance of *TP53* status. The presence of a deletion or mutation of the *TP53* gene in CLL is known to be associated with an unsatisfactory treatment outcome, rapid disease progression, and insensitivity to therapy [11]. Döhner et al. demonstrate the importance of understanding *TP53* status as a reliable prognostic indicator for treatment selection in patients with CLL undergoing conventional therapy [12]. To develop and standardize the study of *TP53* gene alterations in CLL, the ERIC research program was established [13]. In addition, it has been recommended that low-burden *TP53* mutations should not be disregarded in the genetic risk assessment of CML in the era of targeted therapy [14].

Chronic lymphocytic leukemia is the predominant form of leukemia in the United States of America. Based on data from the Surveillance, Epidemiology, and End Results (SEER) database, the yearly incidence rate of chronic lymphocytic leukemia (CLL) is 3.9 cases per 100,000 people. SEER predicts that by 2023, CLL will represent 1% of all newly diagnosed cancer cases in the United States. Approximately 0.6% of adults will be diagnosed with chronic lymphocytic leukemia (CLL) throughout their lifetime.

The majority of cases of chronic lymphocytic leukemia occur in the adult population, with the average age of CLL patients being 70 years. Chronic lymphocytic leukemia shows significant variability in its evolution, with some individuals experiencing a slow progression of the disease, while others experience rapid progression. Survival rates vary from 2 to 20 years, with an approximate average of 10 years. Chronic lymphocytic leukemia is defined by the gradual accumulation of mature cancerous B lymphocytes. The main sites where the disease occurs are the peripheral blood, bone marrow, spleen, and lymph nodes. The disease may present with either an absence of symptoms or the presence of lymphadenopathy, splenomegaly, hepatomegaly, fatigue, fever, night sweats, unintentional weight loss, and early satiety. The diagnosis is made by analyzing the results of flow cytometry and immunophenotyping of peripheral blood. Specifically, it involves the identification of a clonal population of B cells that express CD5, CD19, CD20, CD23, and either κ or λ light chain antigen [15, 16]. The Rai or Binet staging systems are used to assess the clinical stage of the disease, but neither of these approaches can accurately predict the progression of the disease in its early stages. For a more accurate prognosis, it is advisable to include additional biological and genetic markers. For example, deletion of the short arm of chromosome 17 (del[17p]) and/or alterations in the *TP53* gene located on this chromosome indicate resistance to chemoimmunotherapy and decreased disease progression with standard treatment protocols [17].

The International Prognostic Index for Chronic Lymphocytic Leukemia (CLL-IPI) uses genetic, biochemical, and clinical markers to stratify patients into different risk groups. Treatment is not required for all groups; it is often deferred until active symptoms manifest and the Binet or Rai scales indicate an advanced stage. Current treatment protocols offer a range of options for patients who need treatment, including the combination of venetoclax (a B-cell lymphoma 2 (BCL2) inhibitor) and obinutuzumab, targeted treatment with Bruton's tyrosine kinase (BTK) inhibitors such as ibrutinib and acalabrutinib, and chemoimmunotherapy. If it has been more than three years since the last therapeutic intervention, the original course of treatment may be repeated in the event of a relapse. If the disease returns earlier than expected, an alternative treatment plan should be considered. Patients with del (17p) or *TP53* mutations typically exhibit resistance to chemotherapy, making targeted therapy a more effective treatment option for them [18].

This review examines the pivotal role of *TP53* status in shaping treatment strategies for CLL, with a particular emphasis on the emerging potential of CAR-T cell therapy. This study examines the challenges posed by *TP53* dysfunction, a key driver of treatment resistance and poor prognosis, and investigates its intersection with the efficacy of CAR-T therapy. Furthermore, we propose innovative therapeutic approaches, including the incorporation of small molecules specifically designed to target mutant p53 protein, as part of a comprehensive strategy to enhance treatment outcomes for high-risk CLL patients.

## Current Clinical Strategies for Treating CLL

The treatment regimen for chronic lymphocytic leukemia may include different approaches depending on the stage of the disease, the patient's age, and their overall health. Recent advances in the molecular biology of CLL have identified key genetic and molecular factors that significantly impact prognosis and treatment strategies. Mutations in genes such as *TP53*, *NOTCH1*, and *SF3B1*, in conjunction with chromosomal abnormalities like del(17p) and del(11q), are strongly associated with disease progression and resistance to conventional therapies. Furthermore, insights into the role of the B-cell receptor (BCR) signalling pathway have led to the development of targeted agents, such as Bruton's tyrosine kinase (BTK) inhibitors and BCL-2 antagonists, which have significantly impacted the management of CLL [19]. Molecular markers now inform risk stratification and personalised treatment approaches, enabling clinicians to adapt therapies according to the genetic profile of the disease, thereby enhancing outcomes and reducing toxicity.

 The use of kinase inhibitors targeting the BCR pathway (ibrutinib and idelalisib) [20, 21] and the anti-apoptotic protein BCL2 (venetoclax) in the treatment of CLL has yielded great results, especially in patients with *TP53* aberrations [22-24]. Ibrutinib is an inhibitor of Bruton's tyrosine kinase and idelalisib is an inhibitor of the PI3K p110 isoform, both of which are involved in intracellular signaling through multiple receptors, including BCRs. Venetoclax is a BH3 mimetic inhibitor of the anti-apoptotic protein BCL2, which is typically elevated in CLL [25]. Importantly, these drugs have shown equally good results in the treatment of relapsed or refractory CLL, independent of other risk factors that influence the efficacy of chemoimmuno-therapy [25-27]. This makes these drugs attractive treatment options for patients who do not respond to standard therapies.

These drugs have shown remarkable improvements in patients in clinical trials and have subsequently been brought into clinical practice for the treatment of CLL through accelerated approval programs. Currently, ibrutinib is approved in Europe as first-line monotherapy for relapsed/refractory CLL, and in the latter case it is recommended in combination with bendamustine + rituximab [28]. Idelalisib in combination with an anti-CD20 monoclonal antibody (rituximab or ofatumumab) is also indicated for the treatment of relapsed/refractory CLL and as first-line therapy in patients with del(17p)/*TP53* mutations who are not candidates for other therapies [29]. Venetoclax is currently approved in Europe for the treatment of patients with relapsed/refractory CLL who have failed chemotherapy and BCR inhibitor therapy, or patients with del(17p) or *TP53* mutation who are ineligible for or have failed BCR inhibitor therapy [30].

Data from observational studies show that for patients who have discontinued a particular BCR inhibitor due to toxicity, another BCR inhibitor or venetoclax, which is even better tolerated in these patients, may be an alternative option [31, 32]. After one or more unsuccessful therapies, allogeneic hematopoietic stem cell transplantation seems to be an attractive option, especially because new therapies may make patients more suitable for this procedure. Until now, the treatment of people with chronic lymphocytic leukemia who have abnormalities in the *TP53* gene has been limited to evaluation and classification into subgroups. Despite the development of new therapies, patients with *TP53* mutations remain at high risk, but their prognosis has improved significantly over the past decades. More data and additional trials of novel drugs in this cohort are needed to formulate more accurate long-term prognoses. Nevertheless, the aforementioned drugs have already led to a revision of previous guidelines regarding the choice of treatment for patients with CLL. Currently, these drugs, either alone or in various combinations, are the primary treatment for individuals with CLL who have *TP53* abnormalities, as well as for relapsed or resistant CLL [13, 16, 33].

## Prognostic and Therapeutic Significance of TP53 Status in CLL

The *TP53* gene encodes the well-known tumor suppressor protein, p53, which is crucial in pathways that maintain genetic stability and prevent cancer development [34-36]. Additionally, these pathways involve not only p53, but also two closely related proteins p63 and p73 and two negative regulators of p53, MDM2 and MDM4 [37, 38]. The activity of p53 is not limited to its effect on transcription. Transcription-independent functions of p53 have also been described, such as its ability to translocate to mitochondria and exert pro-apoptotic effects through direct interaction with pro- and anti-apoptotic proteins [39]. Transcription-independent functions also fit well with p53's strategy of ensuring genetic stability.

MDM2, a negative regulator, ensures low levels of p53 protein in normal cell conditions. In response to various types of cellular stress, p53 is activated and initiates a series of cellular responses aimed at cellular repair and survival. If repair is not possible, p53 induces cellular growth arrest or programmed cell death [40]. Moreover, recent research has established a link between the function of the p53 protein and the regulation of metabolism and redox equilibrium, both of which play a crucial role in maintaining the internal balance of cells [41].

*TP53* mutations are present in almost all types of cancer, exhibiting a broad array of variability, ranging between 10% to 90%. Notwithstanding divergences like mesothelioma, neuroblastoma, or testicular cancer, *TP53* is still the most frequently altered gene in human cancers [1, 42]. *TP53* mutations in individuals typically indicates an unfavorable prognosis, characterized by more aggressive disease progression, increased recurrence rates, and decreased overall survival. *TP53* mutations in cancer were classified according to their position within the protein, the type of mutation (missense or indel), and the degree of evolutionary conservation of the altered amino acid. The categorization of genetic alterations allowed the assessment of the clinical significance of *TP53* status. The most notable significance was found in head and neck cancer [7], breast cancer [8] or diffuse large B-cell lymphoma [9].

Therapeutic strategies for chronic lymphocytic leukemia (CLL) depend on the presence of *TP53* mutations in the patient and are therefore important examples of the clinical value of TP53 status. Although *TP53* variants are very rare in asymptomatic patients, they are often associated with a poor prognosis characterized by advanced clinical stage, rapid disease progression, chemoresistance, and reduced all-cause mortality (Fig. 2) [12]. 

Given the recent emergence of the clinical significance of *TP53* status, testing for *TP53* mutations has now been incorporated into standard clinical practice. Several commercially available assays can be used to identify point mutations in the *TP53* gene using next-generation sequencing (NGS) or enzyme immunoassay (Table 1). Prior to the implementation of NGS, somatic mutations in the *TP53* gene were primarily observed in the DNA-binding region of the protein, specifically in exons 5-8. Based on these data, the majority of subsequent studies did not consider mutations in other regions of the gene, resulting in a bias [43, 44]. Recently, this bias has been reduced as contemporary research has shifted its attention to the entire coding region of the gene. Collected data, along with advanced research techniques such as next-generation sequencing (NGS), have revealed that exons 2-4 and exons 9-11 of the gene contain approximately 10% of all *TP53* variants. Notably, the range of these variations is different from those previously identified in exons 5-8. These mutations primarily involve insertions or deletions of genetic material, usually resulting in a phenotype termed *TP53*-null [45]. The pathogenicity and clinical utility of these *TP53* gene mutations have been clearly demonstrated during their detection and validation phase. Therefore, screening of exons 2-11 of the *TP53* gene is highly recommended to improve diagnostic accuracy.

Previously, it was thought that the *TP53* gene was expressed as a protein of the same size. However, the expression of the *TP53* gene has been found to have a complex structure and sequence. The existence of multiple p53 isoforms accounts for the broad and diverse effects of the protein on different tissues. To date, researchers have identified at least 12 p53 isoforms produced by alternative translation initiation, alternative promoters, and alternative splicing. All variants of the p53 protein share a common DNA-binding domain but possess distinct trans-activation and inhibitor domains that allow them to exert diverse effects on gene expression. *TP53* uses different mechanisms to transcribe these isoforms [46, 47]. This intricate mechanism of expression suggests that *TP53* intronic sequences do not only form alternative isoforms of protein but may also significantly impact the overall biological functions of p53. Consequently, these sequences may rep-resent critical target regions for somatic or germline variants [48].

The discovery of a hotspot for intron 1 rearrangements further emphasizes the importance of including *TP53* intronic sequences in screening. A recent study found that genetic rearrange-ments in intron 1 are associated with increased cancer risk in four consecutive generations of a family with LFS features. These observations suggest that these genetic abnormalities may predispose individuals to a wide range of malignancies [49]. Rearrangements in intron 1 of the *TP53* gene were identified by Southern blot analysis more than two decades ago. However, at that time, this finding was not considered very sig-nificant and was not incorporated into mutation screening guidelines. The only malig-nancy in which intron 1 rearrangements have been identified is osteosarcoma. Notably, *TP53* missense mutations have long been considered rare in osteosarcoma, and it has been suggested that MDM2 amplification, rather than *TP53* mutations, is the primary mode of p53 protein inactivation in these cancers [50]. In about half of all human osteosarcoma cases, intronic rearrangements were discovered, indicating a significant prevalence of somatic *TP53* mutations in this type of cancer.

## Impact of TP53 Status on Clinical Outcomes in CLL

Several studies [51–55], including results from prospective clinical trials [12, 56, 57], have demonstrated the importance of analyzing *TP53* mutations in CLL. It has become clear that mutations and deletions in the *TP53* gene are associated with resistance to chemoimmuno-therapy (Figures 2, 3) and a negative prognosis for the disease. Therefore, it is currently recommended that patients with CLL be tested for both deletions and mutations in the *TP53* gene prior to treatment in a clinical setting. Accurate assessment of *TP53* gene status is becom-ing increasingly important to identify patients who may be ineligible for chemoimmunotherapy and should instead be considered for targeted or combination therapy. This issue is the focus of numerous clinical trials (Table 2). This is due to the introduction of novel treatment options that inhibit B-cell signaling and the anti-apoptotic BCL2, which have been shown to be effective in patients burdened with *TР53* abnormalities [23, 58, 59].

The diagnosis and evaluation of *TP53* status in chronic lymphocytic leukaemia (CLL) presents a number of challenges that have an impact on treatment outcomes. The detection of *TP53* mutations, such as deletions of 17p or point mutations, is often difficult due to the heterogeneous nature of the mutations, the low allele frequencies, or technical limitations associated with traditional diagnostic methods. The minimum region required for sequencing the *TP53* gene should include the DNA binding domain spanning codons 100-300 and the oligomerization domain corresponding to exons 4-10. To comprehensively analyze the coding sequence of the *TP53* gene, it is necessary to examine exons 2-11 [60]. NGS profiling of the *TP53* gene typically includes exons 2, 3, and 11. Next-generation sequencing provides extensive coverage and allows the identification of mutations in these exons, even if they occur at low frequency. Sequencing of the intronic +2/-2 nucleotides is critical for detecting mutations that may disrupt splicing and result in the production of non-functional proteins, as each exon is flanked by a donor and acceptor splice site.

**Figure 1:**
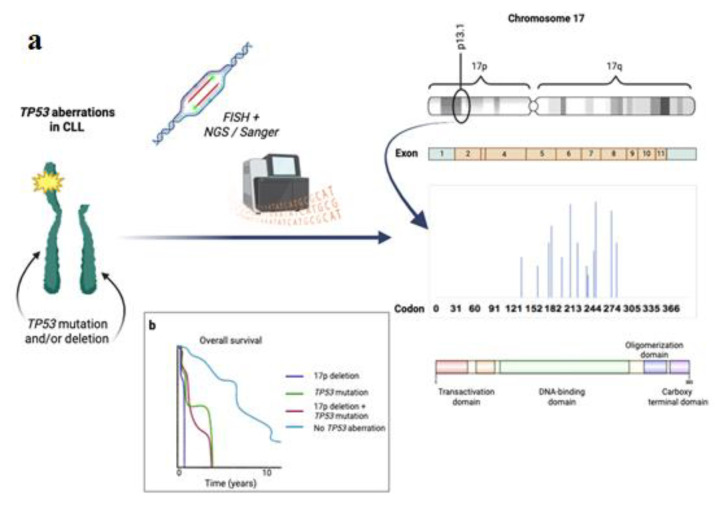
Diagnostics of TP53 mutations.

Incorporation of *TP53* aberrations analysis into routine clinical diagnostics is now standard practice to improve patient classification and maximize treatment options (Fig. 1). The *TP53* gene can be analyzed by bidirectional Sanger sequencing or NGS. In addition, NGS allows the analysis of multiple genes in parallel and has a higher sensitivity threshold, allowing the detection of variants that Sanger sequencing cannot, such as variants with variant allele frequencies (VAF) up to 1% [61–63]. However, NGS currently has a number of technical limitations that can complicate data interpretation. Due to the low detection limit of NGS, multiple subclonal mutations in the *TP53* gene may be detected in some patients.

*TP53* status is one of the most important prognostic and predictive indicators in CLL. With a cutoff of at least 10%, it should be determined using (a) a fluorescence in situ hybridization (FISH) panel to look for del17p13 signatures and (b) a Sanger sequencing or NGS panel to assess *TP53* mutations. Furthermore, it is critical to perform both tests as numerous studies have shown [14,51,52,63,64], that patients with del17p13 alone and those with only a *TP53* gene mutation have equally poor outcomes. Several positions have been identified where the amino acids of the p53 protein are most frequently altered in CLL - 175, 179, 248 and 273 [65]. This suggests that mutations in traditional hotspots are common in CML [66]. 53 out of 268 variants (20%) were found in codons 175, 179, 220, 248, 273 and 281. In addition, another frequently altered codon was found at position 209 (2 nt deletion in all cases). This was the most common alteration together with the hotspots, codons 248 and 273 (Fig. 1).

Targeted therapy has been shown to improve the prognosis of patients with *TP53* mutations of any frequency, suggesting that appropriate genetic screening followed by access to targeted therapy is necessary for patients with *TP53* mutations [67]. In addition, genotoxic chemotherapy has been shown to promote the proliferation of malignant clones, and targeted drugs can limit this process to some extent. Most importantly, this study demonstrated that *TP53* gene mutations remain a significant adverse prognostic factor even in the setting of targeted therapy.

## Emerging Role of CAR-T Therapy in the Treatment of CLL

In 2010, two patients diagnosed with advanced stage CLL participated in a Phase 1 clinical trial that administered tisagenlecleucel, an anti-CD19 chimeric antigen receptor (CAR)-T cell therapy [68]. And even more than a decade after infusion, activated CAR-T cells continued to proliferate in their blood. Over time, the CAR-T cell population in their body changed from predominantly CD8+ to predominantly CD4+. At the same time, the cells remained functionally active and were not depleted, a critical aspect of CAR T-cell therapy for maintaining remission.

CAR-T cell immunotherapy appears to be a promising treatment option for patients with relapsed or refractory high-risk CLL who have not responded well to standard therapy or who have certain genetic abnormalities, such as *TP53* mutation or deletion [69]. The efficacy of CAR-T immunotherapy has been demonstrated in the treatment of many oncohematologic diseases, including B-cell non-Hodgkin's lymphoma and acute lymphoblastic leukemia, and has been approved for use outside of clinical trials [70, 71].

Patients with advanced pretreatment, high-risk, relapsed, or refractory chronic lymphocytic leukemia seem to be very suitable candidates for CAR-T cell therapy. Unfortunately, in most clinical trials of CLL using CAR-T cells, only a minority of patients achieved a complete response (CR) [72]. However, those who did achieve CR with anti-CD19 CAR-T cells experienced long-lasting remissions [73-75]. Sustained remission was associated with an increased levels of CD27+CD45RO-CD8+ T cells prior to CAR-T cells, and these lymphocytes had memory properties [76]. Highly functional CAR-T cells from patients produced STAT3-related cytokines, and serum IL-6 levels correlated with CAR-T cell expansion. In addition, a mechanistically relevant population of CD27+PD-1-CD8+ CAR-T cells expressing high levels of IL-6 receptor predicted therapeutic response and was responsible for tumor control.

The perfect target antigen for CAR-T cells should have high levels of expression on the surface of all tumor cells, maintain complete tumor specificity, and show immunogenicity [77]. However, target selection criteria vary and often need to be modified according to real clinical needs. For example, in the treatment of solid tumors, efficacy improvement is a priority, making the selection of a target with high specificity and coverage crucial. In the treatment of B-lymphomas, however, this is not yet a major obstacle, as CD19 or CD20 have shown to provide sufficient coverage and specificity. On the contrary, the most pressing challenge in the treatment of B lymphoma is in improving the complete remission rate (CRR) and preventing relapse [78].

## CD19

CD19 is a transmembrane protein expressed in all B cells except plasma cells. Even though B-cell tumorigenesis rarely leads to complete CD19 elimination [79, 80], it remains the primary antigen for CAR-T cell therapy in clinical studies targeting chronic lymphocytic leukemia (CLL). More than 100 people with CLL have undergone treatment with anti-CD19 CAR-T cells, but the efficacy of the treatment is lower than expected compared to other B-cell malignancies. Remission rates ranged from 68% to 93% in patients with acute lymphoblastic leukemia (ALL) and 64% to 86% in patients with B-cell lymphoma [81]. Treatment failure is frequently attributed to restricted T-cell expansion and persistence [73, 74, 82], which is commonly observed in patients with large, aggressive nodules [74]. Even though patients with relapsed/persistent CLL were more likely to retain CD19+ following CAR T-cell infusion [73], some cases exhibited escape mechanisms where antigen-negative CD19 relapse occurred [83].

## CD20

CD20 is a transmembrane protein located on the surface of all B cells. It appears during the later phase of pro-B cells and supports the differentiation and specialization of B cells into plasma cells [84]. CD20 has been successfully targeted by monoclonal antibodies such as rituximab, ofatumumab, and obinutuzumab [85], although sustained therapeutic targeting has been associated with downregulation of its expression [86]. Preliminary clinical trials have exhibited promising outcomes when assessing the effectiveness of anti-CD20 CAR-T cell therapy for B-cell lymphoma [87, 88], Additionally, a number of ongoing clinical trials for anti-CD20 CAR-T therapy have enrolled patients with relapsed or refractory CLL (e.g., NCT03277729).

## κ-Immunoglobulin Light Chain

Targeting CAR-T cells with pan-B-cell markers CD19 and CD20 is an effective strategy for achieving long-term control of disease. However, this approach is often associated with B-cell aplasia, which can result in long-term impairment of humoral immunity [87]. The occurrence of such adverse effects has prompted the search for alternative targets. Mature B-lymphocyte immunoglobulins consist of either κ or λ light chain. Malignant B cells exhibit clonal restriction in the expression of κ- and λ- immunoglobulin light chains, resulting in the exclusive expression of either κ or λ by cancer cells [88]. Consequently, CAR-T cells that specifically target the clonally restricted light chain expressed by tumor B-cells would not cause injury to normal B-lymphocytes due to the presence of the contrasting light chain on them. The negative effects on the humoral immunity of patients would be minimized. Promising results have been observed in research conducted both in in vivo and in vitro on CAR-T cells that target the κ-light chain. These cells have demonstrated effectiveness against malignancies expressing the Igκ+ marker [89]. In addition, a phase 1 clinical trial was conducted to evaluate the efficacy of anti-κ CAR-T cell therapy. Two patients with chronic lymphocytic leukemia were enrolled in this study [81]. The patients diagnosed with CLL experienced a prolonged remission from the disease [90].

## CAR-T Therapy for TP53-Mutated CLL

Preclinical studies in mice have shown that *TP53* mutations in CLL not only exacerbate the course of the disease, resulting in high tumor burden, but also have a negative impact on the successful transplantation of T- cells. This ultimately reduces the efficacy of anti-CD19 CAR T-cell therapy (Fig. 3) [91]. In a clinical trial investigating anti-CD19 CAR-T cell therapy, the presence of the del17p gene was detected in twenty patients. Only three of these patients achieved a complete remission [81].

A number of trials are currently underway using different combinations of drugs to treat CLL with *TP53* amplification (Table 2). However, there are no trials of CAR-T therapy and targeted therapy against mutant p53. There are many approaches to restore the lost functions of p53 mutants, including small molecule intervention, gene editing and T-cell immunotherapy [92–95]. Such combination therapy may increase the efficacy of CAR-T therapy in CLL patients with *TP53* mutations.

## CAR-T Cell-Associated Toxicities and Challenges in the Treatment of CLL

The advent of CAR-T therapy has led to significant advances in the treatment of B-cell leukemia and lymphoma but has also been associated with a number of challenges that have the potential to impact the efficacy of the treatment. One significant issue is the condition of the patient's immune system prior to treatment. For instance, patients who have undergone treatment with BTK inhibitors or venetoclax and have subsequently become refractory to these agents may experience diminished efficacy when transitioning to CAR-T therapy, resulting in outcomes that are inferior to those observed in other patients [96]. Furthermore, the efficacy of CAR-T therapy is influenced by the progression of the disease. Patients who experience complications such as Richter's transformation or a high tumour burden tend to respond less favourably to treatment [97,98]. 

Another challenge lies in the severe side effects such as cytokine release syndrome (CRS) and immune cell-associated neurotoxicity syndrome (ICANS) [99]. Cytokine release syndrome results from the hyperactivation of immune cells induced by CAR-T therapy, illustrating the underlying molecular process of this treatment [100]. During anti-CD19 CAR-T cell therapy, cytokine release syndrome occurs as a result of the CAR-T cells activation and proliferation upon recognizing CD19+ target cells. This leads to the release of inflammatory cytokines, such as IL-6, IFN-ɣ, and IL-10 [101], which can cause several symptoms, including fever, vomiting, seizures, and potentially organ failure. Clinical trials involving chronic lymphocytic leukemia (CLL) frequently report CRS as an adverse outcome [75, 102]. The recommended treatment for this condition is corticosteroids and the IL-6 receptor antibody tocilizumab. Typically, even the most severe cases of CRS respond well to this treatment. However, due to the restricted scope of data available, which only accounts for clinical experiences within a small patient cohort, making definitive assertions about the prevalence of this outcome is challenging. Based on the largest collection of research published to date, there is a definitive correlation between the percentage of leukemic B cells present in the bone marrow prior to treatment and the incidence of CRS [74]. Finally, there is evidence that simultaneous use of CAR-T cell and ibrutinib may reduce the incidence of severe CRS [103–105]. This is probably because patients who received this combination therapy had lower levels of inflammatory cytokines [106].

Another side effect observed in clinical trials for CLL is the development of neurological problems, such as cerebral edema. Several recorded cases were classified as severe [106] or even fatal [74]. However, it appears that the onset and severity of the adverse effects noted (cytokine release syndrome and/or neurotoxicity) are similar to those experienced in other hematological conditions, and therefore do not require a distinct dosing regimen for CLL [107].

Studies have shown that ibrutinib can mitigate the side effects of CAR-T therapy [105]. Preclinical studies suggested that ibrutinib can enhance the anti-tumor efficacy of CAR-T cells and reduce CRS, and a pilot study was conducted to assess the safety and feasibility of administering ibrutinib concurrently with CD19 CAR-T cell immunotherapy [108]. 19 CLL patients were enrolled, 17 (89%) of whom had high-risk cytogenetics (17p deletion and/or complex karyotype). In this subgroup, the probabilities of 1-year overall survival and progression-free survival (PFS) were 86% and 59%, respectively. CAR-T cell therapy combined with ibrutinib led to decreased CRS severity and CRS-associated cytokine levels in the serum, as compared to the use of CAR-T cells without ibrutinib. Notably, both treatments exhibited similar in vivo expansion of CAR-T cells. In this study the synergy between CAR-T-19 and ibrutinib mediated a high rate of deep and durable remissions.

## Conclusions and future perspectives

The *TP53* gene plays a pivotal role in maintaining genomic stability and regulating apoptosis, making it a crucial tumor suppressor in cancer biology. Its mutations are among the most frequent genetic alterations in human malignancies, often associated with aggressive disease progression, treatment resistance, and poor clinical outcomes. Despite its well-documented significance, the prognostic value of *TP53* status varies across different cancer types, posing challenges for its consistent integration into clinical decision-making.

In chronic lymphocytic leukemia (CLL), *TP53* alterations, particularly 17p deletions and missense mutations, have emerged as strong predictors of poor response to conventional chemoimmunotherapy. As a result, targeted therapies such as BTK inhibitors (ibrutinib, acalabrutinib), BCL2 inhibitors (venetoclax), and CAR-T cell therapy have been developed to address the limitations of standard treatments in *TP53*-mutated CLL. These novel approaches have shown promising efficacy in high-risk patients, reshaping current treatment paradigms.

CAR-T cell therapy, in particular, has demonstrated potential in achieving durable remissions in relapsed or refractory CLL cases, especially those harboring *TP53* mutations. However, challenges such as suboptimal CAR-T cell persistence, limited response rates, cytokine release syndrome (CRS), and immune exhaustion must be addressed to enhance therapeutic efficacy. The combination of CAR-T therapy with targeted agents, immune checkpoint inhibitors, or gene-editing technologies like CRISPR/Cas9 holds promise for improving patient outcomes. Additionally, bispecific CARs and allogeneic off-the-shelf CAR-T cells are emerging strategies to overcome current limitations.

Looking ahead, several key areas warrant further investigation to optimize *TP53*-targeted strategies in CLL. Standardization of *TP53* testing is essential, with the development of uniform guidelines for detecting mutations and classifying their impact on prognosis to improve clinical decision-making. Simultaneously, personalized treatment strategies should focus on identifying optimal combinations of targeted therapies based on *TP53* status and other molecular markers to ensure more precise and effective approaches. Advancements in CAR-T therapy, including the engineering of more robust and long-lasting CAR-T cells with reduced toxicity and enhanced functionality in *TP53*-mutant settings, represent crucial research priorities. The integration of novel p53-targeting therapies, such as small molecules that restore p53 function or selectively target mutant p53 proteins, offers significant potential for improving outcomes. Finally, long-term monitoring of *TP53*-mutant patients is necessary to understand disease evolution, clonal selection, and resistance mechanisms, ultimately refining therapeutic strategies and improving patient prognosis over time.

With ongoing advancements in molecular oncology and immunotherapy, the integration of *TP53* testing into routine clinical practice will enable more personalized, targeted, and effective treatment strategies for high-risk CLL patients. Continued research and clinical trials will be instrumental in optimizing outcomes and potentially turning *TP53* mutations from a negative prognostic factor into a clinically manageable target.

**Table 1:** Commercial diagnostics kits for detection of mutant *TP53*

**Name**	**Manufacturer**	**Information**
OG-*TP53* kit	SeqPlexing	Detection of point mutations in all coding regions of the *TP53* gene and in exon-ends and intron-ends regions important for splicing.
EasySeq *TP53* Sequencing Kit	NimaGen	NGS library preparation assays based on unique reverse-complement PCR technology for easy, safe and straightforward human gene sequencing.
Ultra-Sensitive *TP53* Mutation Detection Kit	Medaysis	PCR technology capable of detecting common somatic mutations in the *TP53* gene with high specificity and sensitivity.
Elecsys Anti-p53	Roche	In vitro quantitative immunoassay for anti-p53 autoantibodies in human serum and plasma.

**Table 2:** Clinical trials investigating *TP53*-mutated chronic lymphocytic leukemia (CLL)

**Clinical Trial ID**	**Investigational Drug/Combination**	**Study start date**	**Participant Enrollment**
NCT05197192	Acalabrutinib plus Venetoclax plus Obinutuzumab vs Obinutuzumab plus Venetoclax	April 19, 2022	650
NCT04419389	1. APR-246 + Acalabrutinib 2. APR-246 + Venetoclax + Rituximab	March 2, 2021	100
NCT04178798	Acalabrutinib	December 9, 2019	22
NCT04010968	Venetoclax and Ibrutinib Rituximab plus Fludarabine and Cyclophosphamide	September 27, 2019	120
NCT03545035	Idelalisib and Rituximab	February 6, 2019	104
NCT03455517	Venetoclax and Rituximab	October 31, 2018	77
NCT03342144	Venetoclax vs Venetoclax + Rituximab or Obinutuzumab	December 4, 2017	350
NCT03204188	Ibrutinib Fludarabine Pembrolizumab	September 22, 2017	15
NCT02980731	Venetoclax	December 13, 2016	210
NCT02758665	ibrutinib plus venetoclax plus obinutuzumab	September 2016	41
NCT02756611	Venetoclax	June 22, 2016	258
NCT02827617	Ibrutinib	June 1, 2016	56
NCT02232386	Ibrutinib and Rituximab	February 2015	156
NCT02337829	Acalabrutinib	January 12, 2015	48
NCT02264574	Ibrutinib + Obinutuzumab vs Chlorambucil + obinutuzumab	October 6, 2014	229
NCT01659021	Delalisib and Ofatumumab	December 4, 2012	261
NCT01556776	Lenalidomide	July 20, 2012	89
NCT01459211	Lenalidomide & Dexamethasone	May, 2012	12
NCT01678430	Ofatumumab & Chlorambucil vs Ofatumumab & Bendamustine	December 2011	670

Diagnostics of *TP53* mutations. A) Loss of wild-type p53 function in CLL can occur due to del(17p) and/or *TP53* mutations. 17p13 deletion is invariably associated with loss of *TP53*, which is confirmed by fluorescence in situ hybridisation (FISH) and NGS or Sanger. Organization of the *TP53* gene and distribution of mutations by codon in CLL. *TP53* gene is located at p13.1 on short arm of chromosome 17, comprising 11 exons encoding p53 protein. Alterations were identified in nearly all codons of the *TP53* gene, with the majority concentrated in the DNA-binding domain (exons 4-8, codons 100-300). B) Patients with 17p13 deletion or with *TP53* mutation have a poor prognosis.

**Figure 2:**
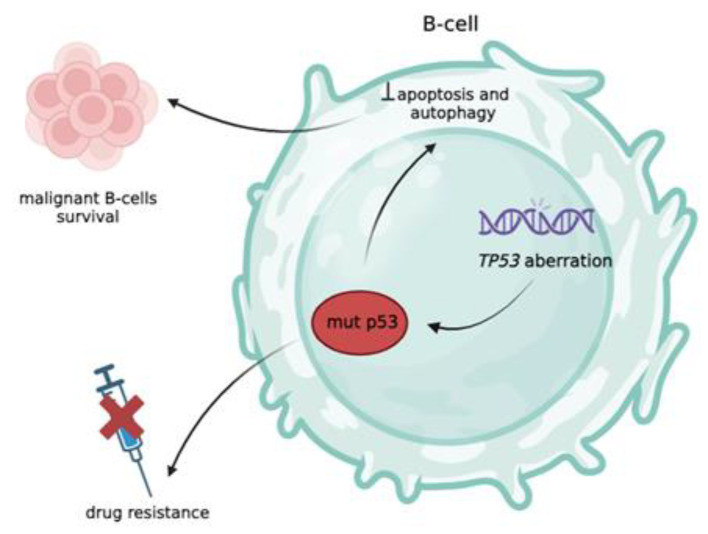
Role of TP53 Aberrations in the Pathogenesis of Chronic Lymphocytic Leukemia (CLL).

*TP53* mutations and deletions disrupt key cellular processes in B-cells, leading to reduced apoptosis and autophagy, which promotes the survival and accumulation of malignant B-cells. The resulting mutant p53 protein impairs the tumor-suppressive functions of wild-type p53, contributing to drug resistance and disease progression. This cascade of events highlights the crucial role of *TP53* aberrations in CLL pathogenesis and their association with poor clinical outcomes.

**Figure 3:**
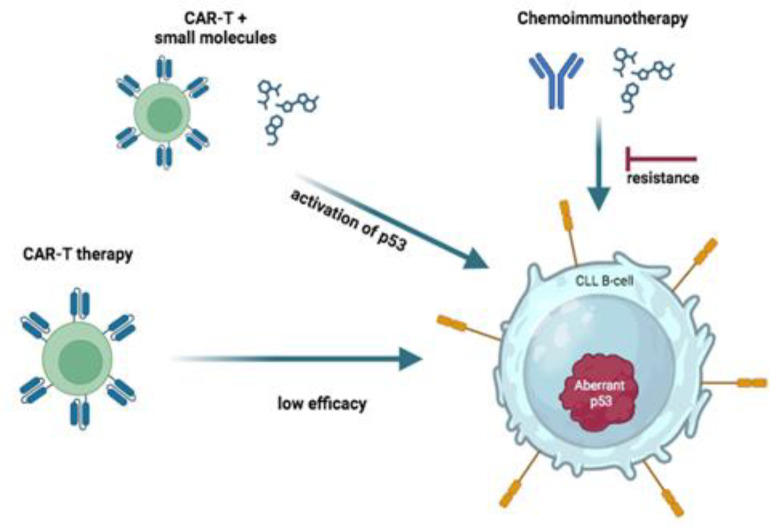
Impact of TP53 Aberrations on Therapeutic Response in Chronic Lymphocytic Leukemia (CLL).

CLL B-cells with aberrant *TP53* exhibit resistance to chemoimmunotherapy, leading to poor clinical outcomes. Traditional CAR-T therapy shows limited efficacy in these cases due to the loss of functional p53. An emerging strategy involves the combination of CAR-T therapy with small molecule inhibitors designed to reactivate p53, potentially improving therapeutic responses. This highlights the need for alternative approaches to overcome treatment resistance in *TP53*-mutated CLL.

## References

[1] Kandoth C, McLellan MD, Vandin F, Ye K, Niu B, Lu C, Xie M, Zhang Q, McMichael JF, Wyczalkowski MA (2013). Mutational landscape and significance across 12 major cancer types. Nature.

[2] Joerger AC, Fersht AR (2010). The tumor suppressor p53: from structures to drug discovery. Cold Spring Harb Perspect Biol.

[3] Romanovsky E, Kluck K, Ourailidis I, Menzel M, Beck S, Ball M, Kazdal D, Christopoulos P, Schirmacher P, Stiewe T, Stenzinger A, Budczies J (2023). Homogenous TP53mut-associated tumor biology across mutation and cancer types revealed by transcriptome analysis. Cell Death Discov.

[4] Robles AI, Harris CC (2010). Clinical outcomes and correlates of TP53 mutations and cancer. Cold Spring Harb Perspect Biol.

[5] Petitjean A, Achatz MIW, Borresen-Dale AL, Hainaut P, Olivier M (2007). TP53 mutations in human cancers: functional selection and impact on cancer prognosis and outcomes. Oncogene.

[6] Bertheau P, Lehmann-Che J, Varna M, Dumay A, Poirot B, Porcher R, Turpin E, Plassa L-F, de Roquancourt A, Bourstyn E, de Cremoux P, Janin A, Giacchetti S, Espie M, de The H (2013). P53 in breast cancer subtypes and new insights into response to chemotherapy. Breast.

[7] Osman AA, Neskey DM, Katsonis P, Patel AA, Ward AM, Hsu TK, Hicks SC, McDonald TO, Ow TJ, Alves MO, Pickering CR, Skinner HD, Zhao M, Sturgis EM, Kies MS, El-Naggar A, Perrone F, Licitra L, Bossi P, Kimmel M, Frederick MJ, Lichtarge O, Myers JN (2015). Evolutionary action score of TP53 coding variants is predictive of platinum response in head and neck cancer patients. Cancer Res.

[8] Olivier M, Langerød A, Carrieri P, Bergh J, Klaar S, Eyfjord J, Theillet C, Rodriguez C, Lidereau R, Bieche I, Varley J, Bignon Y, Uhrhammer N, Winqvist R, Jukkola-Vuorinen A, Niederacher D, Kato S, Ishioka C, Hainaut P, Børresen-Dale AL (2006). The clinical value of somatic TP53 gene mutations in 1,794 patients with breast cancer. Clin Cancer Res.

[9] Young KH, Leroy K, Møller MB, Colleoni GWB, Sánchez-Beato M, Kerbauy FR, Haioun C, Eickhoff JC, Young AH, Gaulard P, Piris MA, Oberley TD, Rehrauer WM, Kahl BS, Malter JS, Campo E, Delabie J, Gascoyne RD, Rosenwald A, Rimsza L, Huang J, Braziel RM, Jaffe ES, Wilson WH, Staudt LM, Vose JM, Chan WC, Weisenburger DD, Greiner TC (2008). Structural profiles of TP53 gene mutations predict clinical outcome in diffuse large B-cell lymphoma: an international collaborative study. Blood.

[10] Leroy B, Ballinger ML, Baran-Marszak F, Bond GL, Braithwaite A, Concin N, Donehower LA, El-Deiry WS, Fenaux P, Gaidano G, Langerod A, Hellstrom-Lindberg E, Iggo R, Lehmann-Che J, Mai PL, Malkin D, Moll UM, Myers JN, Nichols KE, Pospisilova S, Ashton-Prolla P, Rossi D, Savage SA, Strong LC, Tonin PN, Zeillinger R, Zenz T, Fraumeni Jr JF, Taschner PEM, Hainaut P, Soussi T (2017). Recommended guidelines for validation, quality control, and reporting of TP53 variants in clinical practice. Cancer Res.

[11] Gruber M, Wu CJ (2014). Evolving understanding of the CLL genome. Semin Hematol.

[12] Stilgenbauer S, Schnaiter A, Paschka P, Zenz T, Rossi M, Döhner K, Bühler A, Böttcher S, Ritgen M, Kneba M, Winkler D, Tausch E, Hoth P, Edelmann J, Mertens D, Bullinger L, Bergmann M, Kless S, Mack S, Jager U, Patten N, Wu L, Wenger MK, Fingerle-Rowson G, Lichter P, Cazzola M, Wendtner CM, fink AM, Fischer K, Busch R, Hallek M, Dohner H (2014). Gene mutations and treatment outcome in chronic lymphocytic leukemia: results from the CLL8 trial. Blood.

[13] Pospisilova S, Gonzalez D, Malcikova J, Trbusek M, Rossi D, Kater AP, Cymbalista F, Eichhorst B, Hallek M, Döhner H, Hillmen P, van Oers M, Gribben J, Ghia P, Montserrat E, Stilgenbauer S, Zenz T, European Research Initiative on CLL (ERIC) (2012). ERIC recommendda-tions on TP53 mutation analysis in chronic lymphocytic leukemia. Leukemia.

[14] Cherng HJ, Khwaja R, Kanagal‐Shamanna R, Tang G, Burger J, Thompson P, Ferrajoli A, Estrov Z, Sasaki K, Sampath D, Wang X, Kantarjian H, Keating M, Wierda WG, Jain N (2022). TP53 ‐altered chronic lymphocytic leukemia treated with firstline Bruton’s tyrosine kinase inhibitor‐based therapy: A retrospective analysis. Am J Hematol.

[15] Kikushige Y, Ishikawa F, Miyamoto T, Shima T, Urata S, Yoshimoto G, Mori Y, Iino T, Yamauchi T, Eto T, Niiro H, Iwasaki H, Takenaka K, Akashi K (2011). Self-renewing hematopoietic stem cell is the primary target in pathogenesis of human chronic lymphocytic leukemia. Cancer Cell.

[16] Hallek M, Cheson BD, Catovsky D, Caligaris-Cappio F, Dighiero G, Döhner H, Hillmen P, Keating M, Montserrat E, Chiorazzi N, Stilgenbauer S, Rai KR, Byrd JC, Eichhorst B, O'Brien S, Robak T, Seymour JF, Kipps TJ (2018). IwCLL guidelines for diagnosis, indications for treatment, response assessment, and supportive management of CLL. Blood.

[17] Hallek M, Fischer K, Fingerle-Rowson G, Fink AM, Busch R, Mayer J, Hensel M, Hopfinger G, Hess G, von Grünhagen U, Bergmann M, Catalano J, Zinzani PL, Caligaris-Cappio F, Seymour JF, Berrebi A, Jager U, Cazin B, Trneny M, Westermann A, Wendtner CM, Eichhorst BF, Staib P, Buhler A, Winkler D, Zenz T, Bottcher S, Ritgen M, Mendila M, Kneba M, Dohner H, Stilgenbauer S, International group of investigators german chronic lymphocytic leukaemia study group (2010). Addition of rituximab to fludarabine and cyclophosphamide in patients with chronic lymphocytic leukaemia: a randomised, open-label, phase 3 trial. Lancet.

[18] Hampel PJ, Parikh SA (2022). Chronic lymphocytic leukemia treatment algorithm 2022. Blood Cancer J.

[19] Arcari A, Morello L, Borotti E, Ronda E, Rossi A, Vallisa D (2024). Recent advances in the molecular biology of chronic lymphocytic leukemia: How to define prognosis and guide treatment. Cancers (Basel).

[20] O’Brien SM, Lamanna N, Kipps TJ, Flinn I, Zelenetz AD, Burger JA, Keating M, Mitra S, Holes L, Yu AS, Johnson DM, Miller LL, Kim Y, Dansey RD, Dubowy RL, Coutre SE (2015). A phase 2 study of idelalisib plus rituximab in treatment-naïve older patients with chronic lymphocytic leukemia. Blood.

[21] Byrd JC, Brown JR, O’Brien S, Barrientos JC, Kay NE, Reddy NM, Coutre S, Tam CS, Mulligan SP, Jaeger U, Devereux S, Barr PM, Furman RR, Kipps TJ, Cymbalista F, Pocock C, Thornton P, Caligaris-Cappio F, Robak T, Delgado J, Schuster SJ, Montillo M, Schuh A, de Vos S, Gill D, Bloor A, Dearden C, Moreno C, Jones JJ, Chu AD, Fardis M, McGreivy J, Clow F, James DF, Hillmen P, RESONATE Investigators (2014). Ibrutinib versus ofatumumab in previously treated chronic lymphoid leukemia. N Engl J Med.

[22] Seymour JF, Ma S, Brander DM, Choi MY, Barrientos J, Davids MS, Anderson MA, Beaven AW, Rosen ST, Tam CS, Prine B, Agarwal SK, Munasinghe W, Zhu M, Lash LL, Desai M, Cerri E, Verdugo M, Kim SY, Humerickhouse RA, Gordon GB, Kipps TJ, Roberts AW (2017). Venetoclax plus rituximab in relapsed or refractory chronic lymphocytic leukaemia: a phase 1b study. Lancet Oncol.

[23] Stilgenbauer S, Eichhorst B, Schetelig J, Coutre S, Seymour JF, Munir T, Puvvada SD, Wendtner CM, Roberts AW, Jurczak W, Mulligan SP, Bottcher S, Mobasher M, Zhu M, Desai M, Chyla B, Verdugo M, Enschede SH, Cerri E, Humerickhouse R, Gordon G, Hallek M, Wierda WG (2016). Venetoclax in relapsed or refractory chronic lymphocytic leukaemia with 17p deletion: a multicentre, open-label, phase 2 study. Lancet Oncol.

[24] Del Poeta G, Postorino M, Pupo L, Del Principe MI, Dal Bo M, Bittolo T, Buccisano F, Mariotti B, Iannella E, Maurillo L, Venditti A, Gattei V, de Fabritiis P, Cantonetti M, Amadori S (2016). Venetoclax: Bcl-2 inhibition for the treatment of chronic lymphocytic leukemia. Drugs Today (Barc).

[25] Roberts AW, Davids MS, Pagel JM, Kahl BS, Puvvada SD, Gerecitano JF, Kipps TJ, Anderson MA, Brown JR, Gressick L, Wong S, Dunbar M, Zhu M, Desai MB, Cerri E, Enschede SH, Humerickhouse RA, Wierda WG, Seymour JF (2016). Targeting BCL2 with venetoclax in relapsed chronic lymphocytic leukemia. N Engl J Med.

[26] Anderson MA, Tam C, Lew TE, Juneja S, Juneja M, Westerman D, Wall M, Lade S, Gorelik A, Huang DCS, Seymour JF, Roberts AW (2017). Clinicopathological features and outcomes of progression of CLL on the BCL2 inhibitor venetoclax. Blood.

[27] Huber H, Edenhofer S, Estenfelder S, Stilgenbauer S (2017). Profile of venetoclax and its potential in the context of treatment of relapsed or refractory chronic lymphocytic leukemia. Onco Targets Ther.

[28] Woyach JA, Ruppert AS, Heerema NA, Zhao W, Booth AM, Ding W, Bartlett NL, Brander DM, Barr PM, Rogers K, Parikh SA, Coutre S, Lazanski G, Nattam S, Larson RA, Erba HP, Litzow MR, Blachly JS, Owen C, Kuzma C, Abramson JS, Brown JR, Little RF, Smith SE, Stone RM, Mandrekar SJ, Byrd JC (2021). Long-term results of alliance A041202 show continued advantage of ibrutinib-based regimens compared with bendamustine plus rituximab (BR) chemoimmunotherapy. Blood.

[29] Ghia P, Pluta A, Wach M, Lysak D, Kozak T, Simkovic M, Kaplan P, Kraychok I, Illes A, de la Serna J, Dolan S, Campbell P, Musuraca G, Jacob A, Avery E, Lee JH, Liang W, Patel P, Quah C, Jurczak W (2020). ASCEND: Phase III, randomized trial of acalabrutinib versus idelalisib plus rituximab or bendamustine plus rituximab in relapsed or refractory chronic lymphocytic leukemia. J Clin Oncol.

[30] Fischer K, Al-Sawaf O, Bahlo J, Fink AM, Tandon M, Dixon M, Robrecht S, Warburton S, Humphrey K, Samoylova O, Liberati AM, Pinilla-Ibarz J, Opat S, Sivcheva L, Le Du K, Fogliatto LM, Niemann CU, Weinkove R, Robinson S, Kipps TJ, Boettcher S, Tausch E, Humerickhouse R, Eichhorst B, Wendtner CM, Langerak AW, Kreuzer KA, Ritgen M, Goede V, Stilgenbauer S, Mobasher M, Hallek M (2019). Venetoclax and obinutuzumab in patients with CLL and coexisting conditions. N Engl J Med.

[31] Mato AR, Hill BT, Lamanna N, Barr PM, Ujjani CS, Brander DM, Howlett C, Skarbnik AP, Cheson BD, Zent CS, Pu JJ, Kiselev P, Foon K, Lenhart J, Bachow SH, Winter AM, Cruz AL, Claxton DF, Goy A, Daniel C, Issac K, Kennard KH, Timlin C, Fanning M, Gashonia L, Yacur M, Svoboda J, Schuster SJ, Nabhan C (2017). Optimal sequencing of ibrutinib, idelalisib, and venetoclax in chronic lymphocytic leukemia: results from a multicenter study of 683 patients. Ann Oncol.

[32] Jones J, Choi MY, Mato AR, Furman RR, Davids MS, Heffner LT, Cheson BD, Lamanna N, Barr PM, Eradat H, Halwani A, chyla B, Zhu M, Verdugo M, Humerickhouse RA, Potluri J, Wierda WG, Coutre SE (2016). Venetoclax (VEN) monotherapy for patients with chronic lymphocytic leukemia (CLL) who relapsed after or were refractory to ibrutinib or idelalisib. Blood.

[33] Eichhorst B, Robak T, Montserrat E, Ghia P, Hillmen P, Hallek M, Buske C (2015). Chronic lymphocytic leukaemia: ESMO clinical practice guidelines for diagnosis, treatment and follow-up. Ann Oncol.

[34] Harris SL, Levine AJ (2005). The p53 pathway: Positive and negative feedback loops. Oncogene.

[35] Freed-Pastor WA, Prives C (2012). Mutant p53: one name, many proteins. Genes Dev.

[36] Wang SJ, Gu W (2014). To be, or not to be: functional dilemma of p53 metabolic regulation. Curr Opin Oncol.

[37] Candi E, Agostini M, Melino G, Bernassola F (2014). How the TP53 family proteins TP63 and TP73 contribute to tumorigenesis: Regulators and effectors. Hum Mutat.

[38] Stindt MH, Muller PAJ, Ludwig RL, Kehrloesser S, Dötsch V, Vousden KH (2015). Functional interplay between MDM2, p63/p73 and mutant p53. Oncogene.

[39] Green DR, Kroemer G (2009). Cytoplasmic functions of the tumour suppressor p53. Nature.

[40] Hock AK, Vousden KH (2012). Tumor suppression by p53: Fall of the triumvirate?. Cell.

[41] Lee P, Vousden KH, Cheung EC (2014). TIGAR, TIGAR, burning bright. Cancer Metab.

[42] Soussi T, Wiman KG (2015). TP53: an oncogene in disguise. Cell Death Differ.

[43] Takahashi T, Nau MM, Chiba I, Birrer MJ, Rosenberg RK, Vinocour M, Levitt M, Pass H, Gazdar AF, Minna JD (1989). p53: A frequent target for genetic abnormalities in lung cancer. Science.

[44] Nigro JM, Baker SJ, Preisinger AC, Jessup JM, Hostetter R, Cleary K, Bigner SH, Davidson N, Baylin S, Devilee P (1989). Mutations in the p53 gene occur in diverse human tumour types. Nature.

[45] Leroy B, Anderson M, Soussi T (2014). TP53 mutations in human cancer: database reassessment and prospects for the next decade. Hum Mutat.

[46] Bourdon JC, Fernandes K, Murray-Zmijewski F, Liu G, Diot A, Xirodimas DP, Saville MK, Lane DP (2005). p53 isoforms can regulate p53 transcriptional activity. Genes Dev.

[47] Marcel V, Dichtel-Danjoy ML, Sagne C, Hafsi H, Ma D, Ortiz-Cuaran S, Olivier M, Hall J, Mollereau B, Hainaut P, Bourdon JC (2011). Biological functions of p53 isoforms through evolution: lessons from animal and cellular models. Cell Death Differ.

[48] Slatter TL, Hung N, Campbell H, Rubio C, Mehta R, Renshaw P, Williams G, Wilson M, Engelmann A, Jeffs A, Royds JA, Baird MA, Braithwaite AW (2011). Hyperproliferation, cancer, and inflammation in mice expressing a Δ133p53-like isoform. Blood.

[49] Masuda H, Miller C, Koeffler HP, Battifora H, Cline MJ (1987). Rearrangement of the p53 gene in human osteogenic sarcomas. Proc Natl Acad Sci USA.

[50] Oliner JD, Kinzler KW, Meltzer PS, George DL, Vogelstein B (1992). Amplification of a gene encoding a p53-associated protein in human sarcomas. Nature.

[51] Rossi D, Cerri M, Deambrogi C, Sozzi E, Cresta S, Rasi S, De Paoli L, Spina V, Gattei V, Capello D, Forconi F, Lauria F, Gaidano G (2009). The prognostic value of TP53 mutations in chronic lymphocytic leukemia is independent of Del17p13: implications for overall survival and chemorefractoriness. Clin Cancer Res.

[52] Zenz T, Kröber A, Scherer K, Häbe S, Bühler A, Benner A, Denzel T, Winkler D, Edelmann J, Schwänen C, Döhner H, Stilgenbauer S (2008). Monoallelic TP53 inactivation is associated with poor prognosis in chronic lymphocytic leukemia: results from a detailed genetic characterization with long-term follow-up. Blood.

[53] Malcikova J, Smardova J, Rocnova L, Tichy B, Kuglik P, Vranova V, Cejkova S, Svitakova M, Skuhrova Francova H, Brychtova Y, Doubek M, Brejcha M, Klabusay M, Mayer J, Pospisilova S, Trbusek M (2009). Monoallelic and biallelic inactivation of TP53 gene in chronic lymphocytic leukemia: selection, impact on survival, and response to DNA damage. Blood.

[54] Dicker F, Herholz H, Schnittger S, Nakao A, Patten N, Wu L, Kern W, Haferlach T, Haferlach C (2009). The detection of TP53 mutations in chronic lymphocytic leukemia independently predicts rapid disease progression and is highly correlated with a complex aberrant karyotype. Leukemia.

[55] Stengel A, Kern W, Haferlach T, Meggendorfer M, Fasan A, Haferlach C (2017). The impact of TP53 mutations and TP53 deletions on survival varies between AML, ALL, MDS and CLL: an analysis of 3307 cases. Leukemia.

[56] Zenz T, Eichhorst B, Busch R, Denzel T, Häbe S, Winkler D, Bühler A, Edelmann J, Bergmann M, Hopfinger G, Hensel M, Hallek M, Döhner H, Stilgenbauer S (2010). TP53 mutation and survival in chronic lymphocytic leukemia. J Clin Oncol.

[57] Gonzalez D, Martinez P, Wade R, Hockley S, Oscier D, Matutes E, Dearden CE, Richards SM, Catovsky D, Morgan GJ (2011). Mutational status of the TP53 gene as a predictor of response and survival in patients with chronic lymphocytic leukemia: results from the LRF CLL4 trial. J Clin Oncol.

[58] O'Brien S, Jones JA, Coutre SE, Mato AR, Hillmen P, Tam C, Österborg A, Siddiqi T, Thirman MJ, Furman RR, Ilhan O, Keating MJ, Call TG, Brown JR, Stevens-Brogan M, Li Y, Clow F, James DF, Chu AD, Hallek M, Stilgenbauer S (2016). Ibrutinib for patients with relapsed or refractory chronic lymphocytic leukaemia with 17p deletion (RESONATE-17): a phase 2, open-label, multicentre study. Lancet Oncol.

[59] Brown JR, Byrd JC, Coutre SE, Benson DM, Flinn IW, Wagner-Johnston ND, Spurgeon SE, Kahl BS, Bello C, Webb HK, Johnson DM, Peterman S, Li D, Jahn TM, Lannutti BJ, Ulrich RG, Yu AS, Miller LL, Furman RR (2014). Idelalisib, an inhibitor of phosphatidylinositol 3-kinase p110δ, for relapsed/refractory chronic lymphocytic leukemia. Blood.

[60] Lee YH, Song NY, Kim DH, Na HK, Surh YJ (2017). Abstract 1250: Curcumin inhibits migration and growth of human colon cancer cells through covalent modification of oncogenic SIRT1: Cysteine 67 as a potential binding site. Cancer Res.

[61] Malcikova J, Stano-Kozubik K, Tichy B, Kantorova B, Pavlova S, Tom N, Radova L, Smardova J, Pardy F, Doubek M, Brychtova Y, Mraz M, Plevova K, Diviskova E, Oltova A, Mayer J, Pospisilova S, Trbusek M (2015). Detailed analysis of therapy-driven clonal evolution of TP53 mutations in chronic lymphocytic leukemia. Leukemia.

[62] Nadeu F, Delgado J, Royo C, Baumann T, Stankovic T, Pinyol M, Jares P, Navarro A, Martín-García D, Beà S, Salaverria I, Oldreive C, Aymerich M, Suárez-Cisneros H, Rozman M, Villamor N, Colomer D, López-Guillermo A, González M, Alcoceba M, Terol MJ, Colado E, Puente XS, López-Otín C, Enjuanes A, Campo E (2016). Clinical impact of clonal and subclonal TP53, SF3B1, BIRC3, NOTCH1, and ATM mutations in chronic lymphocytic leukemia. Blood.

[63] Rossi D, Khiabanian H, Spina V, Ciardullo C, Bruscaggin A, Famà R, Rasi S, Monti S, Deambrogi C, De Paoli L, Wang J, Gattei V, Guarini A, Foà R, Rabadan R, Gaidano G (2014). Clinical impact of small TP53 mutated subclones in chronic lymphocytic leukemia. Blood.

[64] Döhner H, Stilgenbauer S, Benner A, Leupolt E, Kröber A, Bullinger L, Döhner K, Bentz M, Lichter P (2000). Genomic aberrations and survival in chronic lymphocytic leukemia. N Engl J Med.

[65] Zenz T, Vollmer D, Trbusek M, Smardova J, Benner A, Soussi T, Helfrich H, Heuberger M, Hoth P, Fuge M, Denzel T, Häbe S, Malcikova J, Kuglik P, Truong S, Patten N, Wu L, Oscier D, Ibbotson R, Gardiner A, Tracy I, Lin K, Pettitt A, Pospisilova S, Mayer J, Hallek M, Döhner H, Stilgenbauer S; European Research Initiative on CLL (ERIC) (2010). TP53 mutation profile in chronic lymphocytic leukemia: evidence for a disease specific profile from a comprehensive analysis of 268 mutations. Leukemia.

[66] Khadiullina R, Mirgayazova R, Davletshin D, Khusainova E, Chasov V, Bulatov E (2022). Assessment of thermal stability of mutant p53 proteins via differential scanning fluorimetry. Life (Basel).

[67] Malcikova J, Pavlova S, Kunt Vonkova B, Radova L, Plevova K, Kotaskova J, Pal K, Dvorackova B, Zenatova M, Hynst J, Ondrouskova E, Panovska A, Brychtova Y, Zavacka K, Tichy B, Tom N, Mayer J, Doubek M, Pospisilova S (2021). Low-burden TP53 mutations in CLL: clinical impact and clonal evolution within the context of different treatment options. Blood.

[68] Melenhorst JJ, Chen GM, Wang M, Porter DL, Chen C, Collins MA, Gao P, Bandyopadhyay S, Sun H, Zhao Z, Lundh S, Pruteanu-Malinici I, Nobles CL, Maji S, Frey NV, Gill SI, Loren AW, Tian L, Kulikovskaya I, Gupta M, Ambrose DE, Davis MM, Fraietta JA, Brogdon JL, Young RM, Chew A, Levine BL, Siegel DL, Alanio C, Wherry EJ, Bushman FD, Lacey SF, Tan K, June CH (2022). Decade-long leukaemia remissions with persistence of CD4+ CAR T cells. Nature.

[69] Porter DL, Levine BL, Kalos M, Bagg A, June CH (2011). Chimeric antigen receptor-modified T cells in chronic lymphoid leukemia. N Engl J Med.

[70] Maude SL, Laetsch TW, Buechner J, Rives S, Boyer M, Bittencourt H, Bader P, Verneris MR, Stefanski HE, Myers GD, Qayed M, De Moerloose B, Hiramatsu H, Schlis K, Davis KL, Martin PL, Nemecek ER, Yanik GA, Peters C, Baruchel A, Boissel N, Mechinaud F, Balduzzi A, Krueger J, June CH, Levine BL, Wood P, Taran T, Leung M, Mueller KT, Zhang Y, Sen K, Lebwohl D, Pulsipher MA, Grupp SA (2018). Tisagenlecleucel in children and young adults with B-Cell lymphoblastic leukemia. N Engl J Med.

[71] Locke FL, Ghobadi A, Jacobson CA, Miklos DB, Lekakis LJ, Oluwole OO, Lin Y, Braunschweig I, Hill BT, Timmerman JM, Deol A, Reagan PM, Stiff P, Flinn IW, Farooq U, Goy A, McSweeney PA, Munoz J, Siddiqi T, Chavez JC, Herrera AF, Bartlett NL, Wiezorek JS, Navale L, Xue A, Jiang Y, Bot A, Rossi JM, Kim JJ, Go WY, Neelapu SS (2019). Long-term safety and activity of axicabtagene ciloleucel in refractory large B-cell lymphoma (ZUMA-1): a single-arm, multicentre, phase 1-2 trial. Lancet Oncol.

[72] Bair SM, Porter DL (2019). Accelerating chimeric antigen receptor therapy in chronic lymphocytic leukemia: The development and challenges of chimeric antigen receptor T-cell therapy for chronic lymphocytic leukemia. Am J Hematol.

[73] Porter DL, Hwang WT, Frey NV, Lacey SF, Shaw PA, Loren AW, Bagg A, Marcucci KT, Shen A, Gonzalez V, Ambrose D, Grupp SA, Chew A, Zheng Z, Milone MC, Levine BL, Melenhorst JJ, June CH (2015). Chimeric antigen receptor T cells persist and induce sustained remissions in relapsed refractory chronic lymphocytic leukemia. Sci Transl Med.

[74] Turtle CJ, Hay KA, Hanafi LA, Li D, Cherian S, Chen X, Wood B, Lozanski A, Byrd JC, Heimfeld S, Riddell SR, Maloney DG (2017). Durable molecular remissions in chronic lymphocytic leukemia treated with CD19-specific chimeric antigen receptor-modified T cells after failure of ibrutinib. J Clin Oncol.

[75] Frey NV, Gill S, Hexner EO, Schuster S, Nasta S, Loren A, Svoboda J, Stadtmauer E, Landsburg DJ, Mato A, Levine BL, Lacey SF, Melenhorst JJ, Veloso E, Gaymon A, Pequignot E, Shan X, Hwang WT, June CH, Porter DL (2020). Long-term outcomes from a randomized dose optimization study of chimeric antigen receptor modified T cells in relapsed chronic lymphocytic leukemia. J Clin Oncol.

[76] Fraietta JA, Lacey SF, Orlando EJ, Pruteanu-Malinici I, Gohil M, Lundh S, Boesteanu AC, Wang Y, O'Connor RS, Hwang WT, Pequignot E, Ambrose DE, Zhang C, Wilcox N, Bedoya F, Dorfmeier C, Chen F, Tian L, Parakandi H, Gupta M, Young RM, Johnson FB, Kulikovskaya I, Liu L, Xu J, Kassim SH, Davis MM, Levine BL, Frey NV, Siegel DL, Huang AC, Wherry EJ, Bitter H, Brogdon JL, Porter DL, June CH, Melenhorst JJ (2018). Author Correction: Determinants of response and resistance to CD19 chimeric antigen receptor (CAR) T cell therapy of chronic lymphocytic leukemia. Nat Med.

[77] Wieczorek A, Uharek L (2013). Genetically modified T cells for the treatment of malignant disease. Transfus Med Hemotherapy.

[78] Wei J, Han X, Bo J, Han W (2019). Target selection for CAR-T therapy. J Hematol Oncol.

[79] Scheuermann RH, Racila E (1995). CD19 antigen in leukemia and lymphoma diagnosis and immunotherapy. Leuk Lymphoma.

[80] Uckun FM, Jaszcz W, Ambrus JL, Fauci AS, Gajl-Peczalska K, Song CW, Wick MR, Myers DE, Waddick K, Ledbetter JA (1988). Detailed studies on expression and function of CD19 surface determinant by using B43 monoclonal antibody and the clinical potential of anti-CD19 immunotoxins. Blood.

[81] Mancikova V, Smida M (2021). Current state of CAR T-cell therapy in chronic lymphocytic leukemia. Int J Mol Sci.

[82] Brudno JN, Somerville RP, Shi V, Rose JJ, Halverson DC, Fowler DH, Gea-Banacloche JC, Pavletic SZ, Hickstein DD, Lu TL, Feldman SA, Iwamoto AT, Kurlander R, Maric I, Goy A, Hansen BG, Wilder JS, Blacklock-Schuver B, Hakim FT, Rosenberg SA, Gress RE, Kochenderfer JN (2016). Allogeneic T cells that express an anti-CD19 chimeric antigen receptor induce remissions of B-cell malignancies that progress after allogeneic hematopoietic Stem-cell transplantation without causing graft-versus-host disease. J Clin Oncol.

[83] Cruz CR, Micklethwaite KP, Savoldo B, Ramos CA, Lam S, Ku S, Diouf O, Liu E, Barrett AJ, Ito S, Shpall EJ, Krance RA, Kamble RT, Carrum G, Hosing CM, Gee AP, Mei Z, Grilley BJ, Heslop HE, Rooney CM, Brenner MK, Bollard CM, Dotti G (2013). Infusion of donor-derived CD19-redirected virus-specific T cells for B-cell malignancies relapsed after allogeneic stem cell transplant: a phase 1 study. Blood.

[84] Kozlova V, Ledererova A, Ladungova A, Peschelova H, Janovska P, Slusarczyk A, Domagala J, Kopcil P, Vakulova V, Oppelt J, Bryja V, Doubek M, Mayer J, Pospisilova S, Smida M (2020). CD20 is dispensable for B-cell receptor signaling but is required for proper actin polymerization, adhesion and migration of malignant B cells. PLoS One.

[85] Huhn D, von Schilling C, Wilhelm M, Ho AD, Hallek M, Kuse R, Knauf W, Riedel U, Hinke A, Srock S, Serke S, Peschel C (2001). Emmerich B; German Chronic Lymphocytic Leukemia Study Group Rituximab therapy of patients with B-cell chronic lymphocytic leukemia. Blood.

[86] Hiraga J, Tomita A, Sugimoto T, Shimada K, Ito M, Nakamura S, Kiyoi H, Kinoshita T, Naoe T (2009). Down-regulation of CD20 expression in B-cell lymphoma cells after treatment with rituximab-containing combination chemotherapies: its prevalence and clinical significance. Blood..

[87] Vera J, Savoldo B, Vigouroux S, Biagi E, Pule M, Rossig C, Wu J, Heslop HE, Rooney CM, Brenner MK, Dotti G (2006). T lymphocytes redirected against the kappa light chain of human immunoglobulin efficiently kill mature B lymphocyte-derived malignant cells. Blood.

[88] Fialkow PJ, Najfeld V, Reddy AL, Singer J, Steinmann L (1978). Chronic lymphocytic leukaemia: Clonal origin in a committed B-lymphocyte progenitor. Lancet.

[89] Hudecek M, Schmitt TM, Baskar S, Lupo-Stanghellini MT, Nishida T, Yamamoto TN, Bleakley M, Turtle CJ, Chang WC, Greisman HA, Wood B, Maloney DG, Jensen MC, Rader C, Riddell SR (2010). The B-cell tumor-associated antigen ROR1 can be targeted with T cells modified to express a ROR1-specific chimeric antigen receptor. Blood.

[90] Ramos CA, Savoldo B, Torrano V, Ballard B, Zhang H, Dakhova O, Liu E, Carrum G, Kamble RT, Gee AP, Mei Z, Wu MF, Liu H, Grilley B, Rooney CM, Brenner MK, Heslop HE, Dotti G (2016). Clinical responses with T lymphocytes targeting malignancy-associated κ light chains. J Clin Invest.

[91] Mancikova V, Peschelova H, Kozlova V, Ledererova A, Ladungova A, Verner J, Loja T, Folber F, Mayer J, Pospisilova S, Smida M (2020). Performance of anti-CD19 chimeric antigen receptor T cells in genetically defined classes of chronic lymphocytic leukemia. J Immunother Cancer.

[92] Chasov V, Mirgayazova R, Zmievskaya E, Khadiullina R, Valiullina A, Stephenson Clarke J, Rizvanov A, Baud MGJ, Bulatov E (2020). Key players in the mutant p53 team: Small molecules, gene editing, immunotherapy. Front Oncol.

[93] Chasov V, Zaripov M, Mirgayazova R, Khadiullina R, Zmievskaya E, Ganeeva I, Valiullina A, Rizvanov A, Bulatov E (2021). Promising new tools for targeting p53 mutant cancers: Humoral and cell-based immunotherapies. Front Immunol.

[94] Mirgayazova R, Khadiullina R, Chasov V, Mingaleeva R, Miftakhova R, Rizvanov A, Bulatov E (2020). Therapeutic editing of the TP53 gene: Is CRISPR/Cas9 an option?. Genes (Basel).

[95] Stephenson Clarke JR, Douglas LR, Duriez PJ, Balourdas DI, Joerger AC, Khadiullina R, Bulatov E, Baud MGJ (2022). Discovery of nanomolar-affinity pharmacological chaperones stabilizing the oncogenic p53 mutant Y220C. ACS Pharmacol Transl Sci.

[96] Shadman M, Gauthier J, Khajavian S, Hirayama AV, Lynch RC, Smith SD, Ujjani CS, Chow VA, Kiem HP, Till BG, Gopal AK, Maloney DG, Turtle MBBs C (2019). Relapsed or refractory CLL after CD19-specific CAR-T therapy: Treatment patterns and clinical outcomes. Blood.

[97] Benjamini O, Shimoni A, Besser M, Shem-Tov N, Danylesko I, Yerushalmi R, Tadmor T, Lavie D, Fineman R, Jacobi E, Nagler A, Avigdor A (2020). Safety and efficacy of CD19-CAR T cells in Richter’s transformation after targeted therapy for chronic lymphocytic leukemia. Blood.

[98] Kittai AS, Bond DA, William B, Saad A, Penza S, Efebera Y, Larkin K, Wall SA, Choe HK, Bhatnagar B, Vasu S, Brammer J, Shindiapina P, Long M, Mims A, O'Donnell L, Bhat SA, Rogers KA, Woyach JA, Byrd JC, Jaglowski SM (2020). Clinical activity of axicabtagene ciloleucel in adult patients with Richter syndrome. Blood Adv.

[99] Titov A, Petukhov A, Staliarova A, Motorin D, Bulatov E, Shuvalov O, Soond SM, Piacentini M, Melino G, Zaritskey A, Barlev NA (2018). The biological basis and clinical sym-ptoms of CAR-T therapy-associated toxicites. Cell Death Dis.

[100] June CH, O'Connor RS, Kawalekar OU, Ghassemi S, Milone MC (2018). CAR T cell immunethe-rapy for human cancer. Science.

[101] Teachey DT, Lacey SF, Shaw PA, Melenhorst JJ, Maude SL, Frey N, Pequignot E, Gonzalez VE, Chen F, Finklestein J, Barrett DM, Weiss SL, Fitzgerald JC, Berg RA, Aplenc R, Callahan C, Rheingold SR, Zheng Z, Rose-John S, White JC, Nazimuddin F, Wertheim G, Levine BL, June CH, Porter DL, Grupp SA (2016). Identification of predictive biomarkers for cytokine release syndrome after chimeric antigen receptor T-cell therapy for acute lymphoblastic leukemia. Cancer Discov.

[102] Kochenderfer JN, Dudley ME, Feldman SA, Wilson WH, Spaner DE, Maric I, Stetler-Stevenson M, Phan GQ, Hughes MS, Sherry RM, Yang JC, Kammula US, Devillier L, Carpenter R, Nathan DA, Morgan RA, Laurencot C, Rosenberg SA (2012). B-cell depletion and remissions of malignancy along with cytokine-associated toxicity in a clinical trial of anti-CD19 chimeric-antigen-receptor-transduced T cells. Blood.

[103] Gill S, Vides V, Frey NV, Hexner EO, Metzger S, O'Brien M, Hwang WT, Brogdon JL, Davis MM, Fraietta JA, Gaymon AL, Gladney WL, Lacey SF, Lamontagne A, Mato AR, Maus MV, Melenhorst JJ, Pequignot E, Ruella M, Shestov M, Byrd JC, Schuster SJ, Siegel DL, Levine BL, June CH, Porter DL (2022). Anti-CD19 CAR T cells in combination with ibrutinib for the treatment of chronic lymphocytic leukemia. Blood Adv.

[104] Siddiqi T, Soumerai JD, Wierda WG, Dubovsky JA, Gillenwater HH, Gong L, Mitchell A, Thorpe J, Yang L, Dorritie KA (2018). Rapid MRD-negative responses in patients with relapsed/refractory CLL treated with Liso-Cel, a CD19-directed CAR T-cell product: Preliminary results from transcend CLL 004, a phase 1/2 study including patients with high-risk disease previously treated with ibrutinib. Blood.

[105] Gauthier J, Hirayama AV, Purushe J, Hay KA, Lymp J, Li DH, Yeung CCS, Sheih A, Pender BS, Hawkins RM, Vakil A, Phi TD, Steinmetz RN, Shadman M, Riddell SR, Maloney DG, Turtle CJ (2020). Feasibility and efficacy of CD19-targeted CAR T cells with concurrent ibrutinib for CLL after ibrutinib failure. Blood.

[106] Gauthier J, Hirayama AV, Hay KA, Li D, Lymp J, Sheih A, Purushe J, Pender BS, Hawkins RM, Vakil A, Phi TD, Steinmetz RN, Chapuis AG, Till BG, Dhawale T, Hendrie PC, Kiem HP, Ramos J, Shadman M, Cassaday RD, Acharya UH, Riddell SR, Maloney DG, MBBS Turtle CJ (2018). Comparison of efficacy and toxicity of CD19-specific chimeric antigen receptor T-cells alone or in combination with ibrutinib for relapsed and/or refractory CLL. Blood.

[107] Lemal R, Tournilhac O (2019). State-of-the-art for CAR T-cell therapy for chronic lymphocytic leukemia in 2019. J Immunother Cancer.

[108] Gill S, Vides V, Frey NV, Hexner EO, Metzger S, O'Brien M, Hwang WT, Brogdon JL, Davis MM, Fraietta JA, Gaymon AL, Gladney WL, Lacey SF, Lamontagne A, Mato AR, Maus MV, Melenhorst JJ, Pequignot E, Ruella M, Shestov M, Byrd JC, Schuster SJ, Siegel DL, Levine BL, June CH, Porter DL (2022). Anti-CD19 CAR T cells in combination with ibrutinib for the treatment of chronic lymphocytic leukemia. Blood Adv.

